# The antioxidant Glycitin protects against intervertebral disc degeneration through antagonizing inflammation and oxidative stress in nucleus pulposus cells

**DOI:** 10.18632/aging.205251

**Published:** 2023-11-28

**Authors:** Wei Zhao, Yanpei Li, Xiang Cheng, Hui Wei, Peng Li, Lixia Fan, Kaiwen Liu, Shuai Zhang, Hao Wang

**Affiliations:** 1 Department of Orthopaedic Surgery, Qilu Hospital of Shandong University, Jinan 250012, Shandong, China; 2 Department of Radiology, Qilu Hospital of Shandong University, Jinan 250012, Shandong, China; 3 Cheeloo College of Medicine, Shandong University, Jinan 250012, Shandong, China; 4 Rehabilitation Center, Qilu Hospital of Shandong University, Jinan 250012, Shandong, China; 5 Department of Trauma Orthopaedics, Shandong Provincial Hospital Affiliated to Shandong First Medical University, Jinan 250021, Shandong, China; 6 Department of Anesthesiology, Qilu Hospital of Shandong University, Jinan 250012, Shandong, China

**Keywords:** intervertebral disc degeneration, Glycitin, inflammation, TNF-α, ROS

## Abstract

Intervertebral disc degeneration (IVDD) is a kind of typical degenerative disorder of the skeletal muscle system caused by many factors including aging, abnormal mechanical stress and inflammatory responses. Glycitin is a natural isoflavone extracted from legumes. Previous studies have found that it is anti-inflammatory and promotes wound repair. However, the role of Glycitin in IVDD has not been elucidated. In the present research, we were surprised that Glycitin antagonized the NF-κB pathway activity. In addition, we also found that Glycitin alleviated TNF-α-induced metabolic disorders, extracellular matrix degradation, oxidative stress, inflammation responses, and mitochondrial damage. Furthermore, in *in vivo* experimental study, we discovered Glycitin attenuated IVDD. The results revealed that Glycitin alleviated the degenerative phenotype of IVDD. According to this research, Glycitin has anti-inflammatory properties that might exert a protective function in IVDD, suggesting a prospective therapeutic approach for IVDD.

## INTRODUCTION

Low back pain (LBP) has become the main reason for global productivity decline and the leading reason for disability in most countries [1, 2]. Relevant studies have shown that 80% of adults have suffered from LBP at some point, and the prevalence is higher in older age groups [3]. Intervertebral disc degeneration (IVDD) is receiving increasing attention as a major cause of LBP [4]. The nucleus pulposus (NP) degeneration is key feature of IVDD, manifested by dysfunction of this kind of cells and ECM degradation [5].

The mechanisms of IVDD were not sufficiently clarified. Previous research discovered that the expression of inflammatory factors enhanced in degenerative intervertebral discs, which further accelerated aging and apoptosis of nucleus pulposus cells, and affected the metabolism of ECM [6, 7]. Among the many inflammatory factors involved, tumor necrosis factor (TNF-α) has received much attention because of its position as a predominant pro-inflammatory cytokine and its critical role in the pathogenesis of multiple disease processes [8, 9]. In the degenerative nucleus pulposus, the TNF-α levels were markedly increased, and were positively related to disc degeneration level [10]. Moreover, anti-TNF-α cytokines and drugs exhibit the potential to delay or treatment IVD degeneration [11]. Furthermore, activation of the TNF-α inflammatory pathway in degenerative intervertebral discs further leads to accumulation of lipid reactive oxygen species (Lipid ROS) and activation of oxidative stress responses [12].

Glycitin (C22H22O10) is a physiologically active natural isoflavone produced by soybeans during growth [13]. Previous studies results have shown that Glycitin has a crucial position in the treatment of cardiovascular disease, anti-osteoarthritis, anti-osteoporosis, anti-inflammatory, antioxidant and promoting wound healing [14, 15]. What’s more, Glycitin was discovered to exert an important function in inhibiting the NF-κB pathway in multiple diseases [16].

It is believed that the NF-κB pathway leads to inflammatory factors released in NP cells, promotes the production of inflammatory microenvironment in NP tissue, causes homeostatic imbalance in NP cells, and plays a key role in IVDD [17]. But constricting the NF-κB pathway activity can delay disc degeneration [18, 19]. Nevertheless, the role of Glycitin in IVDD has not yet been clearly evaluated. This research was designed to determine the role and underly molecular mechanism of Glycitin in IVDD.

## MATERIALS AND METHODS

### Media, regents, and animal models

Gibco-BRL (Waltham, MA, USA) provided Dulbecco's Modified Eagle Medium Nutrient Mixture F-12 (#8120482). GibcoBRL (Waltham, MA, USA) provided the fetal bovine serum (#16000044). We used many corresponding antibodies: anti-COX-2 (#ab283574), anti-ADAMTs-5 (#ab231595) from Abcam (Cambridge, UK); anti-NOS-2 (#AF0199), anti-GAPDH (#AF7012), anti-Aggrecan (#AF7561), anti-MMP-13 (#AF5355) and anti-Col-2 (#AF0135) from Affinity Biosciences (Cincinnati, OH, USA); anti-lamin B1 (#17416), anti-Bcl-2 (##3498), anti-Bax (#2772), anti-Cleaved-Caspase-3 (#9661), anti-P65 (#8242), anti-p-P65 (#3033), anti-p-IκBα (#2859), anti-IκBα (#9242) from Cell Signaling Technology (Danvers, MA, USA).

Maintained at Animal Center of Shandong University, all SD rats used in this experiment were 8-12 weeks male rats. The Shandong University Ethics Committee approved all animal studies conducted according to institutional guidelines. All animal experiments were performed according to the guidelines and through the approval of present University.

### Needle puncture model of rat

Based on previously published methods, a model of IVD degeneration was established for needle puncture [20]. In detail, the tail skin was disinfected with iodophor after the rats had been anesthetized. The rats were punctured at 5 mm depth with a 21G needle parallel to the caudal vertebrae. Afterwards, the needle rotated it once and kept at the depth for five seconds. After successful modeling, Glycitin was dissolved in DMSO and diluted to 2 mg/mL with DMEM/F12 basal medium, followed by four weeks of daily treatment with Glycitin (10 mg/kg). To prevent the side effects of intraperitoneal injections, equal dose of DMEM/F12/DMSO was used once a day for 4 weeks in control groups [20].

### Patient samples

Human nucleus pulposus tissue samples were derived from 20 patients (10 males, 10 females). Pfirrmann grading system was adopted to evaluate the degree of intervertebral disc, and Pfirrmann grade II samples were selected for this project. Informed consent was obtained from each donor, and the Medical Ethical Committee of Qilu Hospital of Shandong University approved the project.

### Isolation and culture of primary cells

The cells that were used for this testing were derived from cases that had undergone lumbar spine surgery. The nucleus pulposus sample was cut via sterile scissors and digested with 0.25% trypsin (Gibco, Waltham, MA, USA) for 30 min, then with 0.2% type II collagen (Sigma-Aldrich, St. Louis, MO, USA) for 4 h. NP cells were cultured in complete culture medium consisting of DMEM/F-12 medium, 10% FBS, 1% 100 U/mL penicillin and 100 mg/mL streptomycin (HyClone, Logan, UT, USA). For this experiment, the complete culture medium was changed every 2-3 days, and cells older than three generations were selected after the density reached 80-90%.

### RT-reverse transcriptase-PCR

Human nucleus pulposus cells were collected for examination of transcriptional expression. Total RNA was extracted from the sample via Trizol reagent (R0016, Beyotime, China). RT-PCR kit (Toyota, Japan) was used for cDNA synthesis in accordance with the manufacturers’ instructions. SYBR Green PCR Master Mix (Toyota, Japan) was applied to conduct qRT-PCR and the reaction was performed on a StepOnePlusTM real-time PCR Systems (Eppendorf, Germany). The mRNA level of the target gene was analysed by ΔΔCT and fold changes of mRNA levels were normalized to Glyceraldehyde 3-phosphate dehydrogenase. As shown in Table 1, the relevant primer sequences are listed.

**Table 1:** Primer sequences used for quantitative real-time PCR.

**Primer**	**Forward primers, 5′–3′**	**Reverse primers, 5′–3′**
Homo-ADAMTS5	GAAACAACGGACGCTACTGC	ATGATTTACCATTGGGTGGGCA
Homo-MMP-13	ATTAAGGAGCATGGCGACTTCT	GCCCAGGAGGAAAAGCATGA
Homo-aggrecan	GGTCTCACTGCCCAACTACC	CACGATGCCTTTCACCACGA
Homo-COL2	GATGGCTGCACGAAACATACC	GCCCTATGTCCACACCGAAT
Homo-INOS	CGTGGAGACGGGAAAGAAGT	GACCCCAGGCAAGATTTGGA
Homo-COX2	TCCCTTGGGTGTCAAAGGTAAA	TGGCCCTCGCTTATGATCTG
Homo- CASP3	AAAAGCACTGGAATGACATCTCGG	TGGCTCAGAAGCACACAAACA
Homo-BCL2	GGGTGAACTGGGGGAGGATT	ATCTCCCGGTTGACGCTCTC
Homo-BAX	GAGGTCTTTTTCCGAGTGGCA	GGCAAAGTAGAAAAGGGCGAC
Homo GAPDH	GCACCGTCAAGGCTGAGAAC	TGGTGAAGACGCCAGTGGA

### Western blotting

RIPA lysis buffer (P0013B, Beyotime) and protease inhibitors (P1005, Beyotime) were used to harvest nucleus pulposus cells. The extracted proteins were separated on 10% SDS-polyacrylamide gel and then electrically transferred to the PVDF membrane (FFP24, Beyotime). Following PVDF membrane blockage with 5% BSA at 25° C for 1 hour, it was washed thrice with gentle agitation in TBST at 25° C for 5 min. The PVDF membrane was cultured at 4° C for 16h with relevant primary antibodies. Next we washed the PVDF membrane quartic in TBST at 25° C for 10 min each time. The next steps involve the PVDF membrane being incubated with horseradish peroxidase-conjugated anti-mouse/rabbit secondary antibody (1:5000; Beijing Golden Bridge Biotechnology, China) for 1 hour at 25° C. After washes with TBST, the bound antibody was visualized based on ChemiLuminescence software (Tanon system, Shanghai, China; Amersham Imager 600, GE Amersham, USA). ImageJ software system (Bethesda, MD, USA) was used to analyze the data.

### Immunofluorescence (IF) staining

The sample cells were seeded on 24 well cell inoculum plates. Methanol was used to fix the cells that were subjected to different experimental conditions. The samples were blocked with 5% BSA at 37° C for 30 minutes and then incubated with the specific primary antibody for 1h at 37° C. After three washes with PBS, a secondary antibody conjugated with fluorescein isothiocyanate (1:5000 dilution; Beijing Golden Bridge Biotechnology, China) diluted 1:100 was added for 1h at 37° C. In the next step, nuclei were stained by DAPI and imaged via fluorescence microscope (Ti2-U, Japan).

### Histological staining and histological assessment

The rat sections were stained via Safranin O kit (Beyotime, China), HE staining kit (C0105M, Beyotime) or Masson Staining Kit (GP1032, Servicebio) with the manufacturers’ instructions. An image software IX71-SIF microscope was applied to collect image data.

According to the literature scoring criteria, the staining results were scored histologically. In simple terms, the classification was carried out according to the number and morphology of AF and NP cells, and boundary of them. The overall grades ranged from 5 to 15, with 1 point for each type of normal disc and 15 points for the most severe degeneration [8].

### TUNEL assay

The TUNEL assay kit (C1088; Beyotime) was used to stain adherent cells in accordance with the manufacturers’ instructions and detect the cell apoptosis in each experimental group.

### JC-1 assay

The JC-1 assay kit (C2005, Beyotime) was applied to stain adherent cells in accordance with the manufacturers’ instructions and detect the mitochondrial membrane potential.

### ROS assay

The ROS assay kit (S0033M, Beyotime) was applied to stain adherent cells in accordance with the manufacturers’ instructions and detect ROS levels.

### Flow cytometry

Flow cytometry was used to test whether Glycitin affected TNF-α-induced apoptosis. The sample cells were seeded on 6 well plates. After adding DMSO (control group), Glycitin (100 μg/ml) overnight, cells were stained according to manual specifications (E-CK-A211, Elabscience, USA). Then the samples were detected at CytoFLEXS flow cytometer (Beckman Company, Brea, CA, USA) and CtyExpert software was applied to treat the data.

### X-ray and MRI analyses

Rats in each experimental group accepted X-ray and MRI scans after experimental treatment. After anesthesia, the rats were kept in the prone position with tails upright and X-ray and MRI detection were conducted. For the X-ray images, IVD height and nearby vertebral body heights were detected via the ImageJ tool, and to determine disc height index (DHI). The MRI images were analyzed through Pfirrmann grading method.

### P65 nuclear translocation

The sample cells were treated with or without Glycitin (100 μg/ml) overnight followed by 6 hours treatment with TNF-α (10 ng/mL). Immunofluorescence staining was applied to explore the location of p65 level in the cells. Further the nucleus pulposus cytoplasmic and nuclear protein fractions were extracted through Nuclear and Cytoplasmic Protein Extraction Kit (P0027, Beyotime). p65, Lamin B1 and GAPDH were detected by Western blot.

### Statistical analysis

Data were expressed as means ± standard errors of the mean (SEM). Differences between two or among multiple groups were checked via Student’s t-test or one-way analysis of variance respectively. The Prism software 8.0 (GraphPad Inc, San Diego, CA, USA) and SPSS 17.0 (SPSS Inc, Chicago, IL, USA) were used to conduct the statistical testing. P<0.05 was regarded as the statistical difference threshold.

### Availability of data and materials

The datasets used and/or analyzed of this study are available from authors on reasonable request.

## RESULTS

### Glycitin alleviated the TNF-α-induced inflammatory response

**Figure 1:**
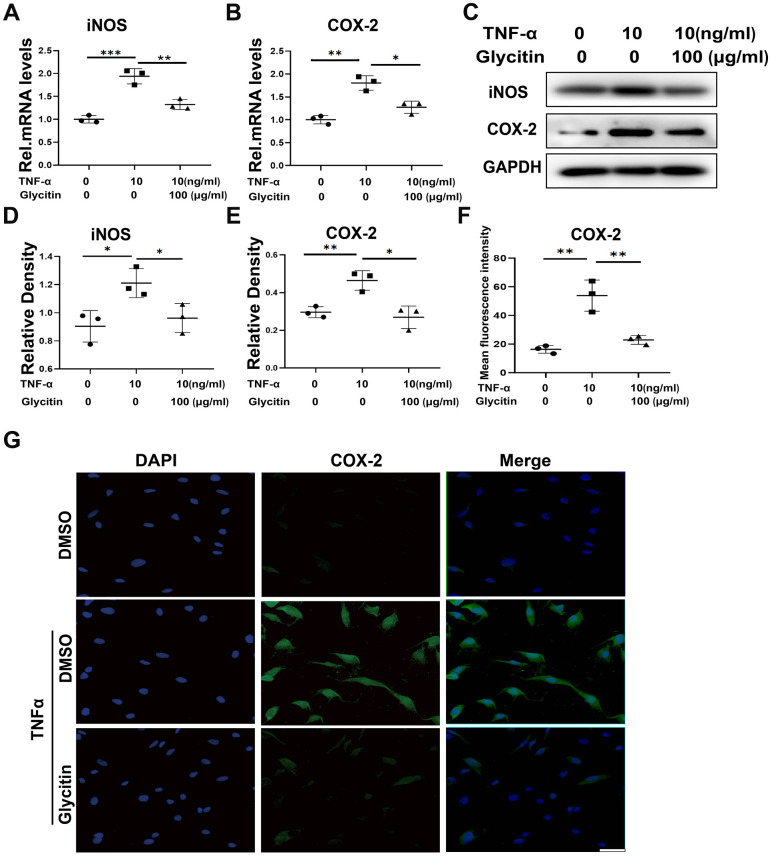
**Glycitin alleviated the TNF-α-induced inflammatory response.** (**A**, **B**) Transcriptional and (**C**) protein levels of iNOS and COX-2 in human primary nucleus pulposus cells as determined by RT-PCR and Western blotting. (**D**, **E**) Quantitative analysis of immunoblotting in (**C**), assayed by ImageJ program. (**F**, **G**) IF staining of COX-2, fluorescence intensity analysis was performed using ImageJ program. Scale bar: 100 μm. The values represent the mean ± SD of three independent experiments. *p < 0.05 vs. control group.

The high level of inflammatory cytokines, particularly TNF-α, is a key factor in IVDD process [21]. Hence, we investigated the impact of Glycitin on IVDD progression by subjecting HNPCs to different treatments for 24 hours to assess mRNA levels and for 48 hours to assess protein levels. These treatments included DMSO, TNF-α (10 ng/ml), and TNF-α (10 ng/ml) + Glycitin (100 μg/ml). The results depicted in Figure 1A–1C, reflected through RT-PCR and immunoblotting analysis, demonstrated a significant upregulation in the mRNA and protein expression of proinflammatory molecules such as iNOS and COX-2 in the presence of TNF-α. But the expression of relevant molecules was significantly reduced upon Glycitin treatment. Furthermore, statistical analysis further confirmed the notable difference (Figure 1D, 1E). Additionally, Figure 1F, 1G presented the immunofluorescence staining, illustrating the expression of COX-2. This staining exhibited that Glycitin treatment effectively mitigated the TNF-α -induced inflammation of HNPCs.

### Glycitin retained ECM metabolism of HNPCs in stimulation of TNF-α

**Figure 2:**
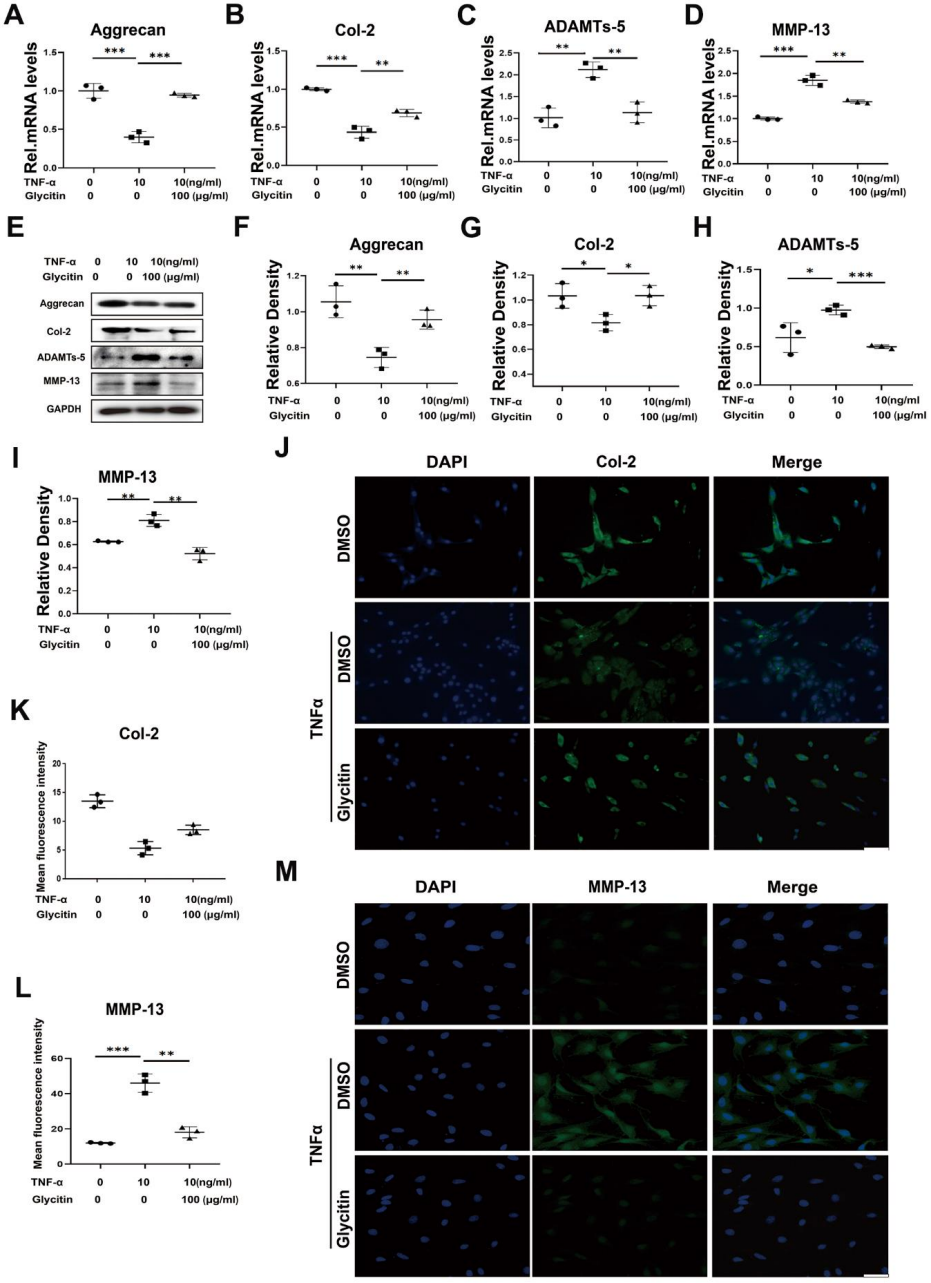
**Glycitin restrained ECM metabolism and alleviated.** (**A**–**D**) Transcriptional and (**E**) protein levels of Aggrecan, Col-2, ADAMTs-5 and MMP-13 in cells as determined by RT-PCR and WB method. (**F**–**I**) Quantitative analysis of immunoblotting in (**E**), assayed by ImageJ program. (**J**–**M**) IF staining of COX-2, fluorescence intensity analysis was performed using ImageJ program. Scale bar: 100 μm. The values represent the mean ± SD of three independent experiments. *p < 0.05 vs. control group.

The nucleus pulposus tissue consists predominantly of type II collagen and Aggrecan [22]. The degradation of components within the extracellular matrix is a critical characteristic of intervertebral disc degeneration [23]. Previous studies have indicated that TNF-α promotes the upregulation of ADAMTSs and matrix metalloproteinases (MMPs), resulting in the breakdown of ECM and the inhibition of ECM anabolism [24]. To analyse the role of Glycitin in maintaining ECM homeostasis in degenerative intervertebral discs, this study cultured and stimulated HNPCs with DMSO, TNF-α (10 ng/ml), or TNF-α (10 ng/ml) + Glycitin (100 μg/ml) for 24 h to assess mRNA levels and for 48 h to determine protein levels. The results depicted in Figure 2A–2D show that TNF-α stimulation led to a decline in the mRNA expression levels of anabolic biomarkers Col-2 and Aggrecan, while elevating the catabolic biomarkers MMP-13 and ADAMTS-5 expression. Interestingly, the additional use of Glycitin significantly altered these trends. Figure 2E–2I demonstrate that Glycitin reversed the TNF-α-induced decrease in Aggrecan, Col-2 protein level and reduced the protein level of MMP-13 and ADAMTS-5. Importantly, as Figure 2J–2M, our studies were further verified by IF staining.

### Glycitin attenuated TNF-α-induced HNPCs apoptosis in HNPCs

**Figure 3:**
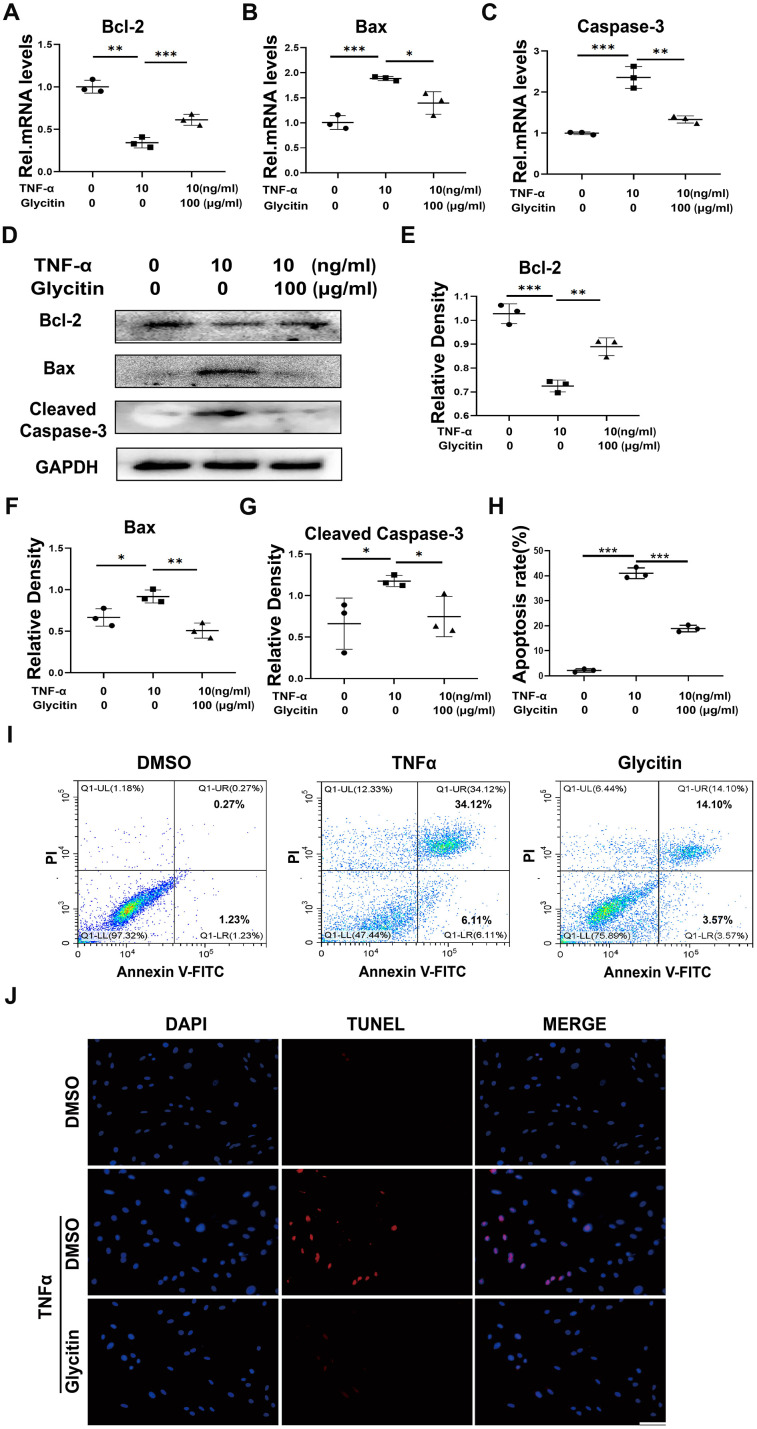
**Glycitin alleviated the TNF-α-induced HNPCs apoptosis.** (**A**–**C**) Transcriptional and (**D**) protein levels of Bcl-2, Bax and Cleaved Caspase-3 in human primary nucleus pulposus cells as determined by RT-PCR and WB method. (**E**–**G**) Quantitative analysis of immunoblotting in (**D**), assayed by ImageJ program. (**H**, **I**) Quantification of NP cell apoptosis by flow cytometry. (**J**) TUNEL staining of the human NP cells of every group (n=3). Nuclei were stained with DAPI. Scale bar, 100 μm. The values shown represent the mean ± SD of three independent experiments. *p < 0.05 vs. control group.

Previous studies have indicated that the progression of IVDD involves the crucial role of apoptosis in nucleus pulposus cells [25, 26]. This paper researched the potential of Glycitin in treating apoptosis in HNPCs, the cells were incubated with TNF-α, with or without Glycitin for 24h and mRNA levels of apoptosis-related molecules were measured. As depicted in Figure 3A–3C, the transcriptional level changes of Bax, Caspase-3, and Bcl-2 induced by TNF-α were observed to be restored by the inclusion of Glycitin. Additionally, the cells underwent the indicated treatments for 48 hours to assess the expression of apoptosis indexes expression at the protein levels. The Western blot analysis demonstrated the inhibitory function of Glycitin on TNF-α-caused apoptosis in HNPCs (Figure 3D–3G).

To gain further insights into the therapeutic role of Glycitin in HNPCs apoptosis, the flow cytometry (Figure 3H, 3I) and the TUNEL assay (Figure 3J) were employed. These measurements provided evidence that Glycitin significantly reversed TNF-α-mediated cell apoptosis.

### Glycitin improved TNF-α-induced oxidative stress and mitochondrial dysfunction in HNPCs

**Figure 4:**
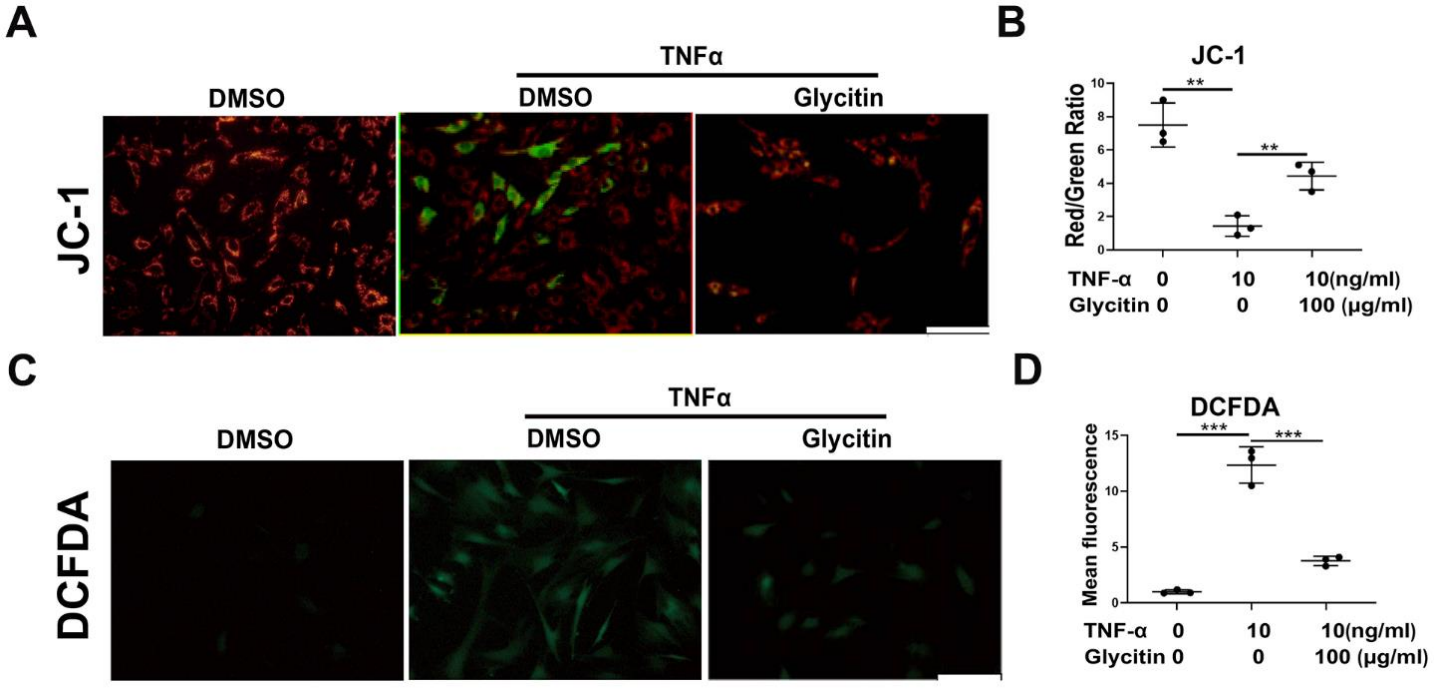
**Glycitin alleviated TNF-α-induced oxidative stress and mitochondrial dysfunction in HNPCs.** (**A**, **B**) JC-1 assay was applied to detect the mitochondrial membrane potential of human NP cells in every group (n=3). Scale bar: 100μm. (**C**, **D**) ROS levels of human NP cells were detected with DCFDA (n=3). Scale bar: 100 μm. The values shown represent the mean ± SD of three independent experiments. *p < 0.05 vs. control group.

It is known that exacerbation of oxidative stress and dysfunction of mitochondria are closely associated with IVD degeneration [27–29]. To analysis the potential inhibitory effect of Glycitin on that in HNPCs, this study cultured HNPCs with DMSO, TNF-α (10 ng/ml), TNF-α (10 ng/ml) + Glycitin (100 μg/ml). In this study, as shown in Figure 4A, JC-1 staining was conducted to measure the mitochondrial dysfunction by membrane potential, and we found the dysfunction which resulted by TNF-α were alleviated after treatment with Glycitin. Furthermore, to assess the influence of Glycitin on mitochondrial ROS during IVDD, DCFDA staining assays were employed, as depicted in Figure 4B. The outcomes revealed the efficacy of Glycitin in protecting against disc degeneration in HNPCs by inhibiting TNF-α-mediated mitochondrial dysfunction.

### Glycitin protected against the degeneration of IVD in rats *in vivo*

**Figure 5:**
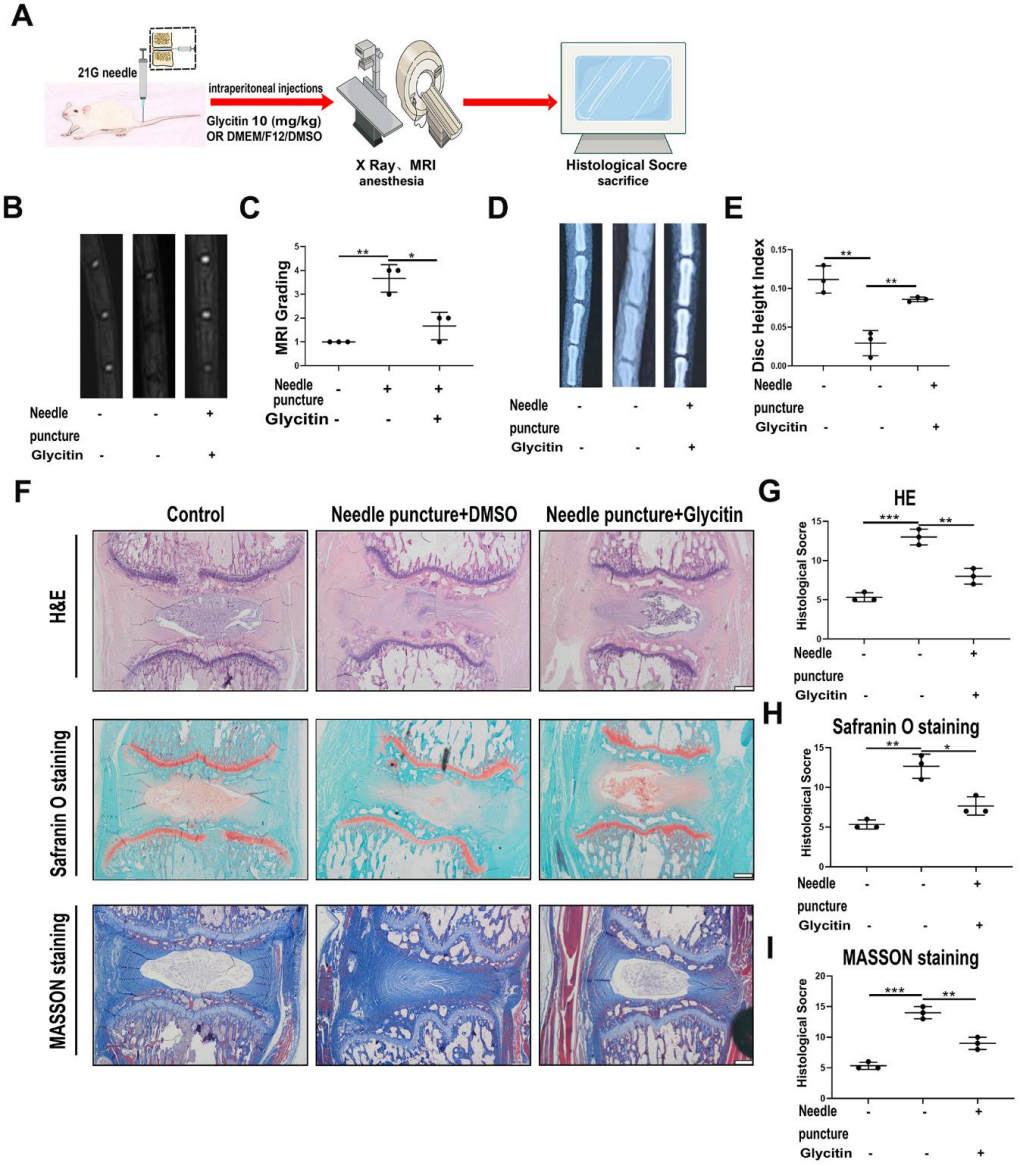
**Glycitin alleviated the degeneration of IVD in rats *in vivo*.** (**A**) Schematic diagram of needle puncture model of rat. (**B**, **C**) MRI image was used to assess the signal strength of IVDD in rats from every group (n = 3). (**D**, **E**) X-ray was applied to evaluate the height of the intervertebral space in rats from each group (n = 3). (**F**–**I**) HE, Safranin-O and Masson staining were conducted to evaluate the IVDs in rats from every group (n=3). Histological score was obtained from staining data. Scale bar: 100 μm. The values represent the mean ± SD of three independent experiments. *p < 0.05 vs. control group.

To further assess the attenuation of IVDD by Glycitin, we utilized the rat IVD needle puncture model. The rats were subjected to intraperitoneal administration for 4 weeks, as depicted in Figure 5A. As Figure 5B, 5C, X-ray indicated the IVD height was obviously lost in the model group. But Glycitin effectively reversed such trend. Moreover, MRI-T2WI result indicated that Glycitin obviously prevented signal intensity loss (Figure 5D, 5E). Thereafter, Safranin O staining and Masson staining were employed to evaluate cartilage tissue loss induced by needle puncture injury, and Glycitin exhibited a rescuing effect in preventing cartilaginous tissue destruction (Figure 5F, 5H, 5I). Further examination through H&E staining demonstrated the detrimental impact of needle puncture injury on the tissue structure of the IVD (Figure 5F, 5G). Conversely, the additional use of Glycitin effectively restored the structural damage caused by the injury.

### Glycitin antagonized the activation of the NF-κB signaling pathway

**Figure 6:**
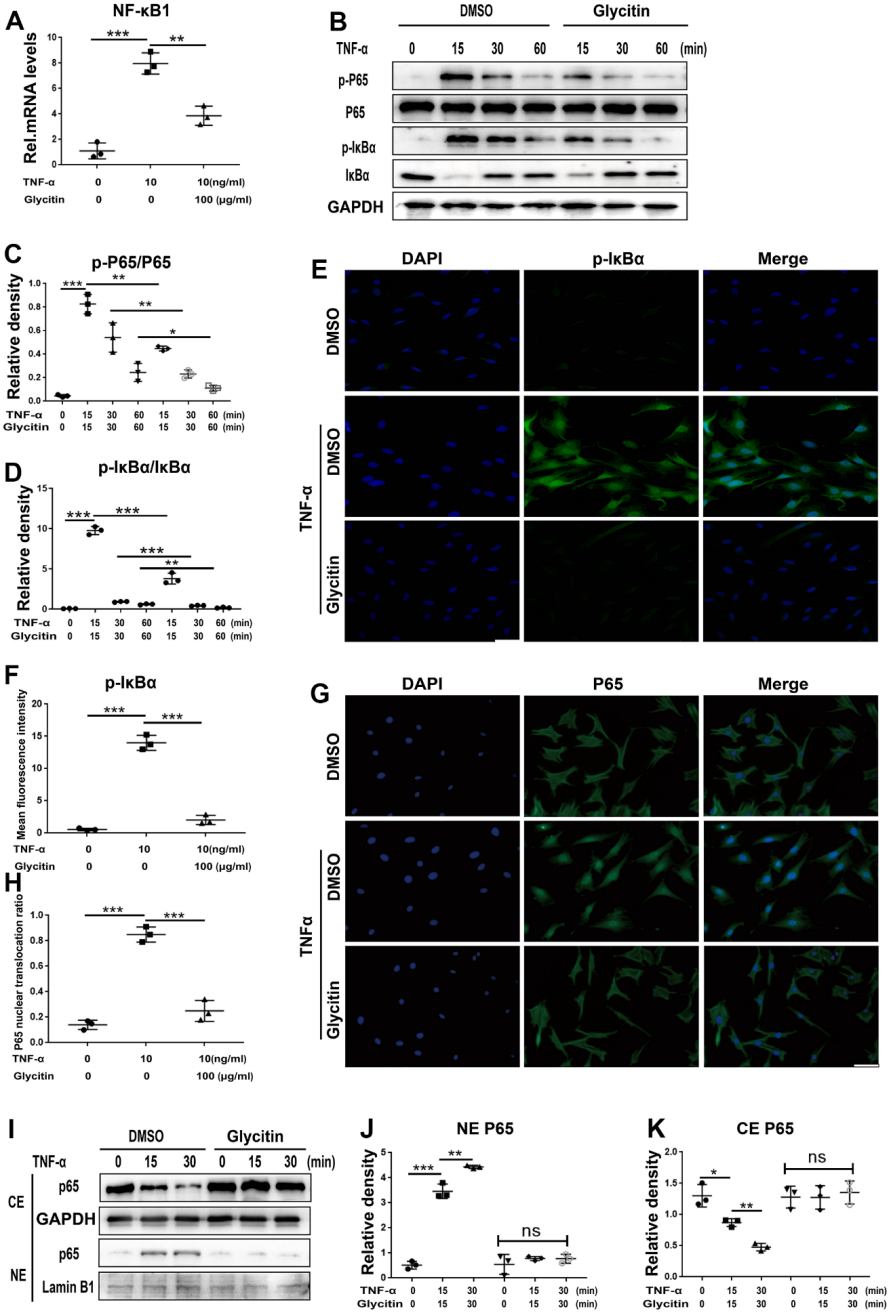
**Glycitin antagonized the activation of the NF-κB signaling pathway.** (**A**) The NF-κB1 level was assayed via RT-PCR. (**B**–**D**) Protein levels of pIκBα and p-P65 were tested by WB method using image J. (**E**, **F**) Immunofluorescence to detect pIκBα in HNPCs of each indicated group (n=3). Scale bar, 100 μm. (**G**, **H**) The HNPCs were treated with or without Glycitin (100 μg/ml) for 12 hours, and then adding TNF-α (10 ng/mL) for 6 h. Immunofluorescence cell staining was applied to determine the p65 location. Scale bar: 100 μm. (**I**–**K**) Nuclear translocation of p65 were determined by WB analysis using ImageJ. The values represent the mean ± SD of three independent experiments. *p < 0.05 vs. control group.

Numerous studies suggested a strong correlation between IVDD and the NF-κB pathway [30, 31]. To analyse the potential ability of Glycitin to prevent the TNF-α/NF-κB pathway, we cultured and stimulated HNPCs using TNF-α, and following treatment with or without Glycitin, total cellular mRNA was extracted. As indicated in Figure 6A, we found that Glycitin effectively hindered the activation of NF-κB. Thereafter, at different time points, we collected total cellular proteins, the Western blot showed that Glycitin completely suppressed this kind of phosphorylation of IκBα and p65 at indicated different time points (Figure 6B–6D). In addition, the TNF-α-mediated increase in pIκBα expression levels was abolished by Glycitin, as shown by IF staining in Figure 6E, 6F.

To further validate the Glycitin intervention on NF-κB pathway, the translocation of p65 was analyzed by IF staining (Figure 6G, 6H) and Western blotting analysis (Figure 6I–6K). Our findings indicate that TNF-α largely increased the translocation level of p65 to the nucleus. Interestingly, the addition of exogenous use of Glycitin reversed TNF-α-mediated P65 translocation.

## DISCUSSION

Numerous studies suggested that nucleus pulposus cells in IVDD can produce and secrets a variety of inflammatory factors, like IL-1β, IL-6, iNOS, TNF-α [7, 22, 32]. TNF-α is related to many pathological variations of IVDD and it is considered as a crucial element of the inflammatory cascade, and its levels are positively correlated to IVDD severity [33]. TNF-α can also lead to increased catabolism of IVD by inducing the level of MMPs and ADAMTSs, disrupting the balance of the myeloid environment, exacerbating ECM degradation, and further accelerating IVDD [34]. Additionally, TNF-α can stimulate NP cells to generate a number of proinflammatory cytokines, thereby intensifying the inflammatory responses [12]. Moreover, TNF-α was implicated in disc herniation and nerve irritation and in growth. It was indicated that a key role of TNF-α in the progression of IVDD [35, 36]. As a result, TNF-α is a potential target for clinical intervention of IVDD. In present research, we stimulated nucleus pulposus cells with TNF-α to reveal the function and mechanisms of Glycitin in preventing IVDD. Glycitin is a natural isoflavone isolated from plants. The safety of Glycitin is our primary concern, and we have gone through a series of experiments to ensure its safety in cells and mice (Supplementary Figure 1).

Compared with the DMSO group, TNF-α effectively caused the inflammatory response, significantly increasing the level of inflammatory factors, including COX-2 and iNOS. Interestingly, the inflammatory response caused by TNF-α was inhibited after therapy with Glycitin compared with the TNF-α induce group, which reflected that Glycitin may alleviate the inflammatory level of degenerative intervertebral discs. In addition, TNF-α leads to increased expression of destructive matrix components (MMP-13 and ADAMTS-5), resulting in significantly reduced expression of extracellular matrix components, including Aggrecan and Col-2. In this study, Glycitin effectively reversed the TNF-α-mediated loss of Col-2 and Aggrecan, and reduced the level of MMP-13 and ADAMTS-5, suggesting that Glycitin is a key role in metabolic disorders of IVD.

Previous research confirmed that TNF-α can promote apoptosis in nucleus pulposus cells [37, 38]. The imbalance of Bax and Bcl-2 level induced C-caspase-3 expression and caused NP cells apoptosis, which are related to the mitochondrial apoptosis pathway during intervertebral disc degeneration. According to the current study, Glycitin treatment decreased Bax and C-caspase-3 level, while Bcl-2 expression enhanced. It is implied that Glycitin reduces the increase in TNF-α -mediated apoptosis of HNPCs cells.

Some scholars found that mitochondrial dysfunction plays a significant role in IVDD development [39]. Our study found that HNPCs after TNF-α treatment had increased ROS expression levels and impaired mitochondrial membrane potential than the control group. Many scholars found that there is a crosstalk between ROS and TNF-α [40]. ROS production leads to oxidative stress, which is very important in the inflammatory process [41]. ROS can activate various transcription factors, causing differential expression of many genes related to the inflammatory pathway [42]. NF-κB is a typical transcription factor [43]. And ROS translocates the active NF-κB to nucleus, where it induces the expression of abundant molecules, like TNF-α, IL-1β and IL-6, which are molecules associated with the inflammatory response of IVDD [44]. Our findings suggest that Glycitin significantly reversed these changes.

To further validate the therapeutic effect of Glycitin in IVDD, we designed *in vivo* experiments to investigate. By establishing the needle puncture rat IVDD model, we found that the results of needle puncture injury included alteration of disc signal, reduction of disc height and loss of cartilage [45]. The results showed that Glycitin has a protective function in IVDD and exhibited a treatment effect in the rat IVD needle puncture model.

**Figure 7:**
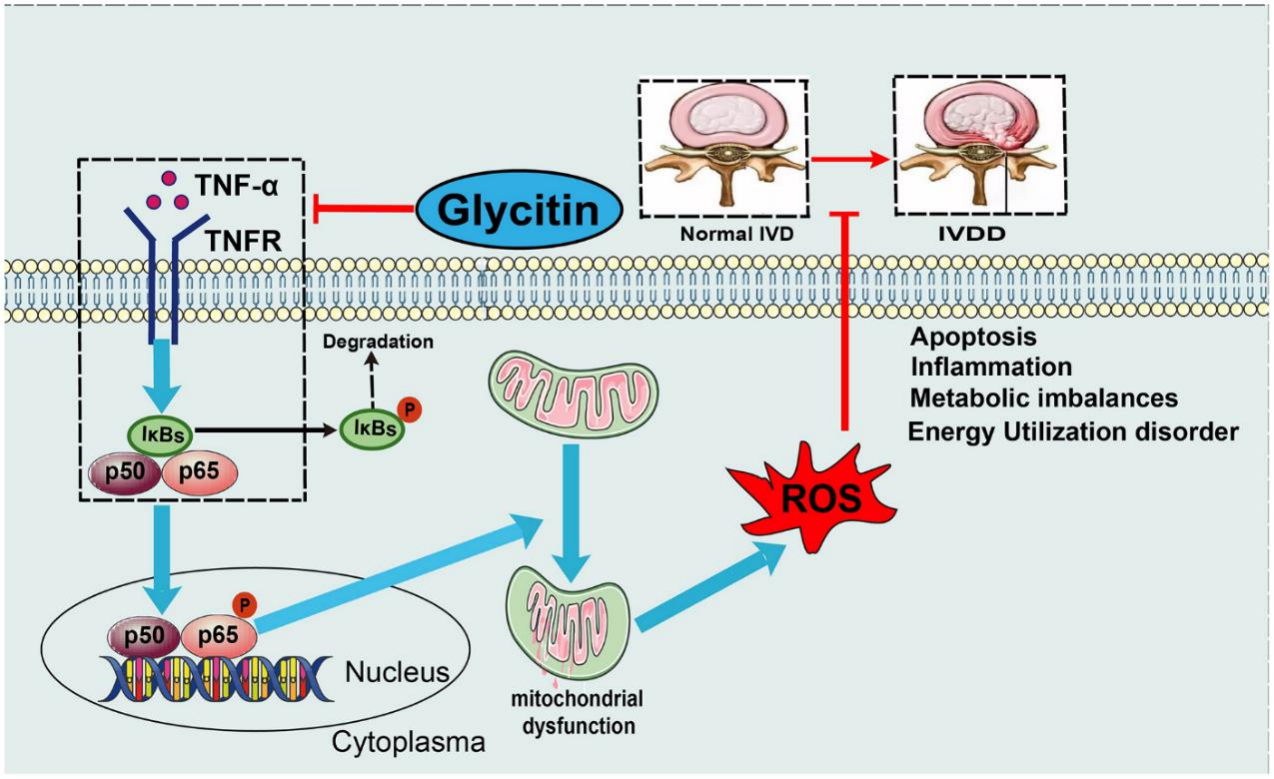
**Model diagram.** A proposed model for depicting the function of Glycitin in IVDD.

In addition, the NF-κB signaling pathway is a classic downstream inflammatory pathway activated by TNF-α and is closely associated with the development of multiple inflammatory degenerative diseases [46]. In intervertebral disc degeneration, activation of the TNF-α/NF-κB signaling pathway leads to the secretion of inflammatory factors from nucleus pulposus cells, promoting the generation of an inflammatory microenvironment in nucleus pulposus tissue and causing a homeostatic imbalance in the nucleus pulposus tissue internal environment [5, 47]. It is widely believed that activation of the NF-κB signaling pathway is related to IVDD; activation of this pathway leads to accelerated disc degeneration, whereas blocking this signaling pathway activation alleviates the aging process [48]. TNF-α, mainly by activating the NF-κB pathway, is related to various pathological processes of intervertebral disc degeneration [12]. By detecting the level of mRNA, the results indicated that the Glycitin inhibited the TNF-α-induced increase in NF-κB1 level. TNF-α increases the levels of key parameters of the NF-κB signaling pathway, including pIκB and p-P65 protein. Intriguingly, the expression levels of pIκBα and p-P65 were remarkably decreased by Glycitin therapy. Moreover, the translocation of p65 indicates the activation of the NF-κB signaling pathway. However, Glycitin abolished P65 nuclear translocation. Glycitin appears to be beneficial in inhibiting TNF-α/NF-κB signaling in IVDD (Figure 7). Further, we showed that Glycitin is comparable to SN50 in inhibiting the NF-κB signaling pathway in IVDD and is a potential NF-κB pathway inhibitor (Supplementary Figure 2). However, it is unclear which molecules were targeted by Glycitin during this process, which will be the topic of our next studies. In conclusion, the findings from *in vivo* and *in vitro* suggest that Glycitin has great potential in the treatment of IVDD.

## Supplementary Material

Supplementary Figures
