# Supplementary material for: The antioxidant Glycitin protects against intervertebral disc degeneration through antagonizing inflammation and oxidative stress in nucleus pulposus cells
Source: Aging (Albany NY). 2023 Nov 28;15(23):13693–709. doi: 10.18632/aging.205251 (PMC10756108; doi:10.18632/aging.205251)
Supplement: Supplementary Figures [file aging-15-205251-s001.pdf]

SUPPLEMENTARY FIGURES

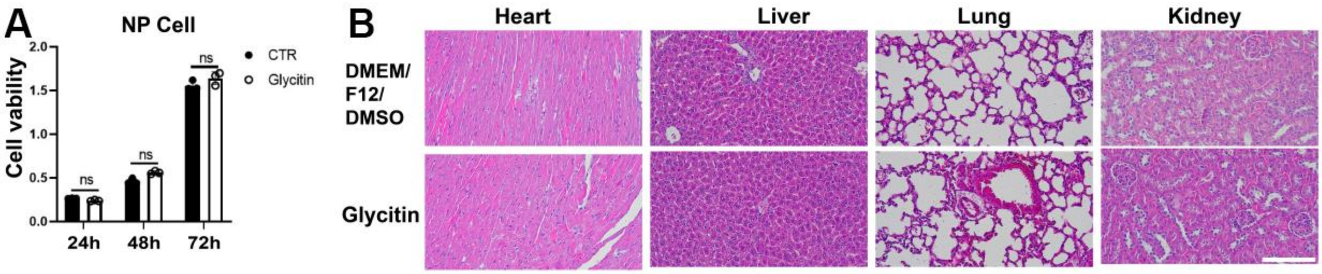

Supplementary Figure 1. Safety of Glycitin *in vitro* and *in vivo*. (**A**) The hNP cells were cultured and treated with Glycitin between 24h, 48h and 72 h. Cell viability was measured using the CCK8 assay in hNP cells. (**B**) The heart, liver, lung, and kidney were isolated from rat treated with Glycitin for 4 weeks. (Scale bar = 500 μm). All data are expressed as means ± SD. *p < 0.05, **p < 0.01, ***p <0.001, ****p < 0.0001 versus control group.

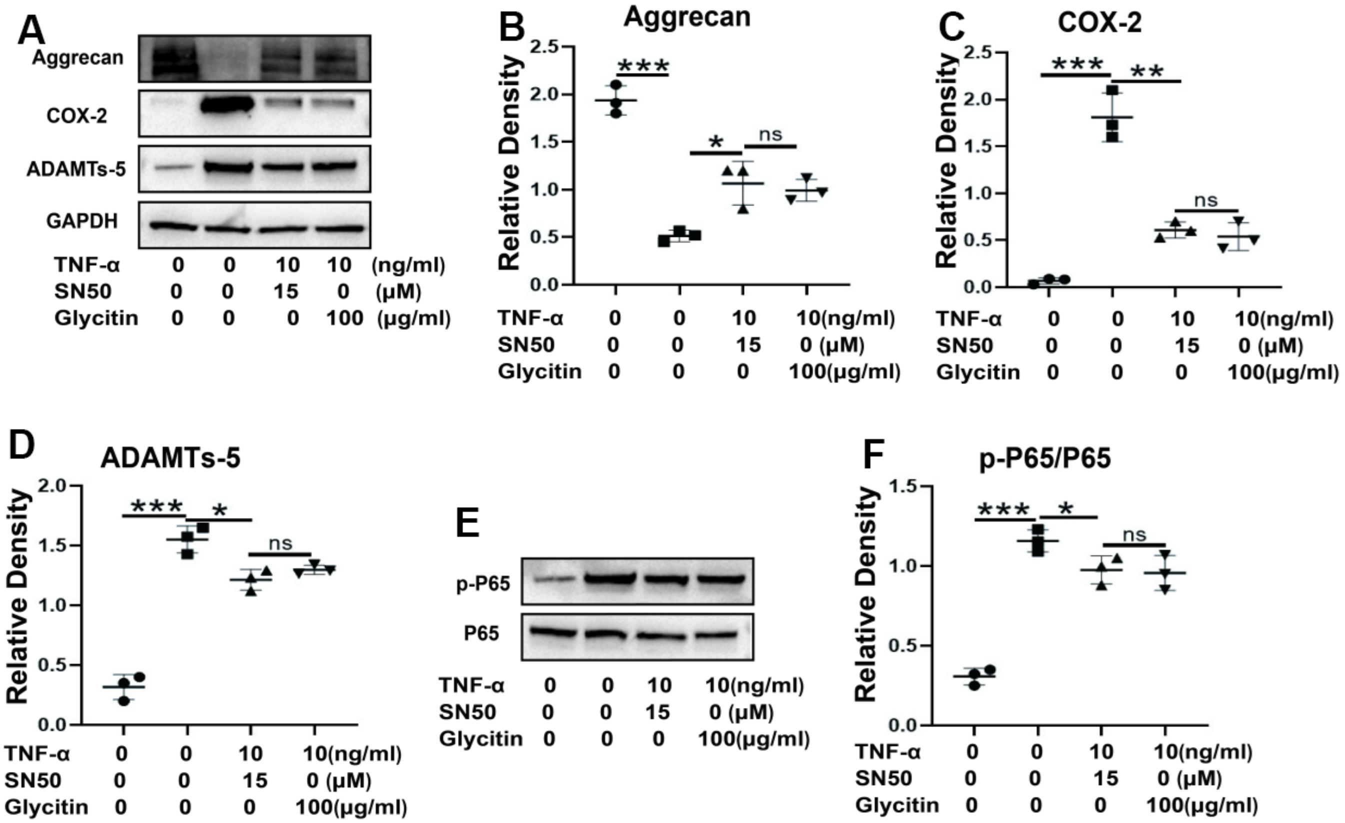

Supplementary Figure 2. Glycitin mimics the role of SN50 in inhibition of the NF-κB signalling pathway. (**A**–**D**) HNPCs were treated with DMSO, TNF-α (10 ng/ml), TNF-α (10 ng/ml) + SN50 (15 μM) and TNF-α (10 ng/ml) + Glycitin (100 μg/ml) 48 hours. Aggrecan, COX-2, ADAMTs-5 were tested by Western blot. The expression of proteins mentioned above were quantitatively analyzed related to GAPDH (n=3). (**E**, **F**) HNPCs were treated with DMSO, TNF-α (10 ng/ml), TNF-α (10 ng/ml) + SN50 (15 μM) and TNF-α (10 ng/ml) + Glycitin (100 μg/ml) for 24 hours. Western blot shows the expression of p-P65 and p65. The expression of proteins mentioned above was quantitatively analyzed (n=3). All data are expressed as means ± SD. *p < 0.05, **p < 0.01, ***p <0.001, ****p < 0.0001 versus control group.
